# Minimization of proteome reallocation explains metabolic transition in hierarchical utilization of carbon sources

**DOI:** 10.1128/msystems.00690-25

**Published:** 2025-06-30

**Authors:** Zhihao Liu, Minghao Chen, Jingmin Hu, Yonghong Wang, Yu Chen

**Affiliations:** 1 State Key Laboratory of Bioreactor Engineering, East China University of Science and Technology 47860, Shanghai, China; 2 State Key Laboratory of Quantitative Synthetic Biology, Shenzhen Institute of Synthetic Biology, Shenzhen Institutes of Advanced Technology, Chinese Academy of Sciences, Shenzhen, China; Northwestern University Feinberg School of Medicine, Chicago, Illinois, USA

**Keywords:** enzyme constraint, metabolic model, mixed carbon sources, metabolic transition

## Abstract

Cells choose between alternative pathways in metabolic networks under diverse environmental conditions, but the principles governing the choice are insufficiently understood, especially in response to dynamically changing conditions. Here, we observed that the lactic acid bacterium *Bacillus coagulans* displayed homolactic fermentation on glucose or trehalose as the sole carbon source but transitioned from homolactic to heterolactic fermentation during the hierarchical utilization of glucose and trehalose when growing on the mixture. We simulated the observation by dynamic minimization of reallocation of the proteome (dMORP) using an enzyme-constrained genome-scale metabolic model, which coincided with our omics data. Moreover, we evolved strains to co-utilize mixed carbon sources and repress the choice of heterolactic fermentation, and the dynamics after co-utilization of carbon sources were also captured by dMORP. Altogether, the findings suggest that upon environmental changes, bacteria tend to minimize proteome reallocation and accordingly adjust metabolism, and dMORP would be useful in simulating cellular dynamics.

**IMPORTANCE:**

Redundancy in metabolic networks empowers cells to choose between distinct metabolic strategies under changing environments. However, what drives the cellular choice remains poorly understood. We hypothesized that in response to rapid environmental changes, cells might minimize reallocation of the proteome and accordingly adjust metabolism. We found that this hypothesis could interpret a metabolic transition in the lactic acid bacterium *Bacillus coagulans* during the hierarchical utilization of glucose and trehalose, which was validated using systems biology approaches. Furthermore, we presented a framework with the objective function of minimizing proteome allocation, allowing for the simulation and understanding of cellular responses to dynamic perturbations.

## INTRODUCTION

Cells have alternative pathways, i.e., redundancy, in metabolic networks to adapt to diverse environmental conditions. For instance, while many microbes and mammalian cells use respiration for ATP production, some of them also harbor fermentation as an alternative ATP-producing pathway, which enables not only survival under anaerobic conditions but also fast growth in the presence of oxygen, i.e., aerobic fermentation (1). The alternative pathways empower cells to choose between distinct metabolic strategies, but the principles governing the cellular choice are insufficiently understood (2).

Experimental and theoretical studies suggest that the choices of alternative pathways and metabolic strategies reflect trade-offs, mostly between metabolic efficiency (e.g., yield) of pathways and cellular resources (e.g., proteome) invested in enzymes of the pathways (3–8). For instance, fermentation has a lower ATP yield but is more proteome efficient than respiration and, therefore, favors fast-growing cells subject to the limited proteome resources (9, 10). These studies are predominantly focused on steady-state cultures, e.g., chemostats, of model microbes, and therefore, the cellular choice might be a result of a relatively long-term adaptation to the environment. However, much less is known about whether and how cells can transition between alternative pathways under dynamically changing environments, which are ubiquitous in both natural and artificial biological systems (11, 12).

*Bacillus coagulans* is a lactic acid bacterium widely used in industrial lactate fermentation (13–15), where hydrolysates of low-cost starch-based materials are commonly used as carbon sources (16, 17), considering cost and benefit. However, the co-existence of multiple carbon sources in the medium may lead to hierarchical utilization (18–23), resulting in the waste of substrates (24), extended fermentation cycles (25), and difficulties in product separation and purification (26). Therefore, identifying the principles governing the dynamic behavior of *B. coagulans* during the hierarchical utilization of mixed carbon sources would be of significance from both basic and applied science perspectives.

Here, we studied *B. coagulans* DSM 1 = ATCC 7050, which has alternative pathways for catabolizing glycolytic carbon sources (e.g., glucose) and generating energy, i.e., homolactic and heterolactic fermentation (13, 27). The former converts glucose to lactate via the Embden–Meyerhof–Parnas pathway, with the theoretical yield being 1 g of lactate per gram of glucose, while the latter converts glucose via the phosphoketolase pathway with a halved yield due to the production of acetate and ethanol (28, 29). We observed that *B. coagulans* displayed homolactic fermentation on glucose or trehalose as the sole carbon source but transitioned from homolactic to heterolactic fermentation in the hierarchical utilization of glucose and trehalose when growing on the mixture. Currently, typical objective functions (e.g., maximization of growth, product formation, or non-growth-associated maintenance) are widely employed for dynamic simulations (30–32). However, these objective functions are not applicable to our study. Therefore, we hypothesized that the metabolic transition could be interpreted by minimization of reallocation of proteome (MORP) and performed enzyme-constrained metabolic modeling and omics analysis to test the hypothesis. Finally, by adaptive laboratory evolution (ALE), we evolved strains that can co-utilize the mixed carbon sources, which showcased another application of MORP.

## RESULTS

### Lactate yield significantly changed in hierarchical utilization of glucose and trehalose

**Fig 1:**
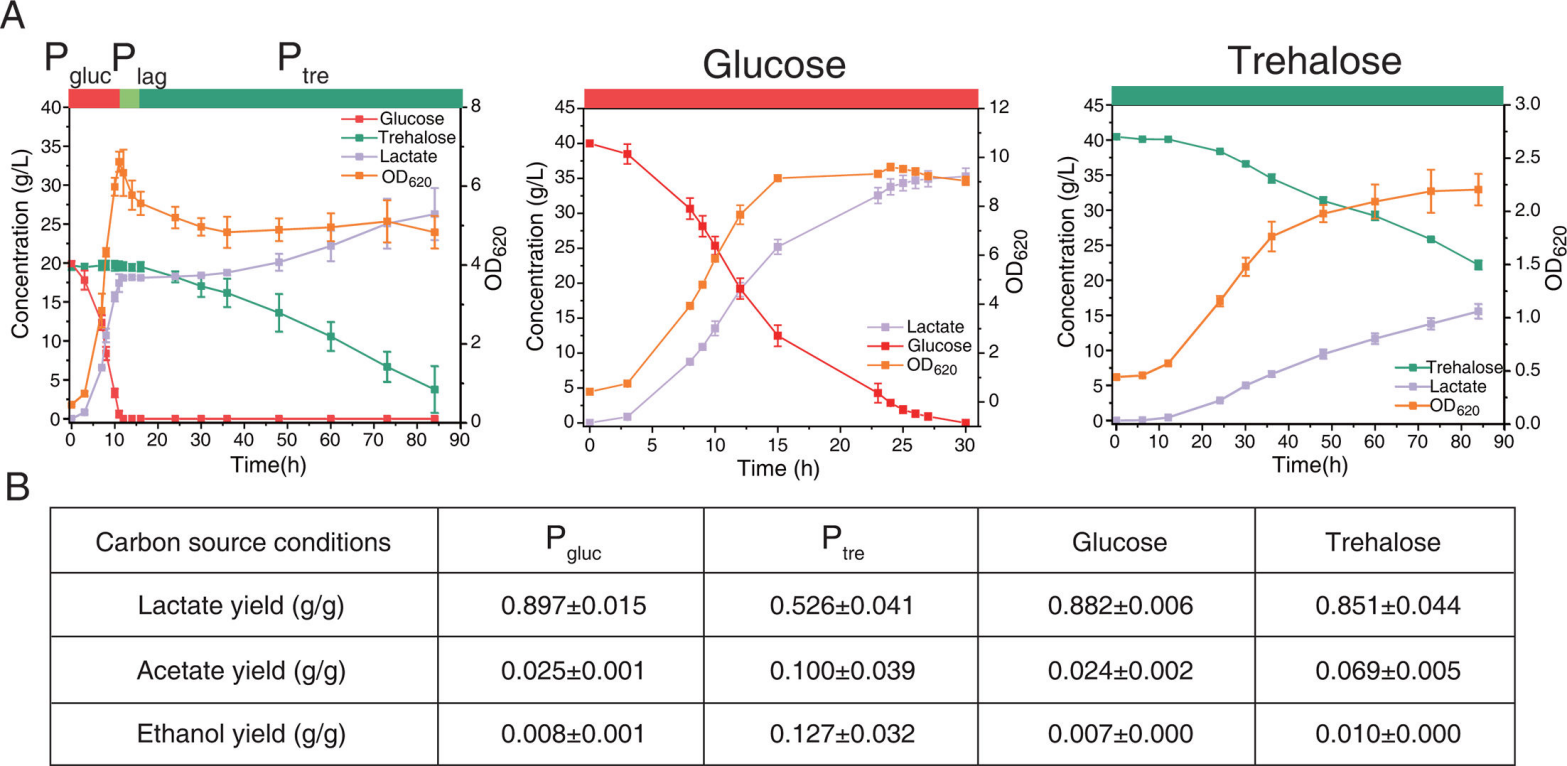
Fermentation on various carbon sources in a 5 L bioreactor by *B. coagulans*. (**A**) Fermentation processes on glucose and trehalose as the mixed carbon sources (left), sole glucose (middle), and sole trehalose (right). Data in the plots are shown with mean ± s.d. of three biological replicates. (**B**) Yields of products under various phases and conditions. The yields for *P*_gluc_ and single glucose conditions are based on glucose, and those for *P*_tre_ and single trehalose conditions are based on trehalose. Data in the table are shown with mean ± s.d. of three biological replicates.

To investigate the principles governing the dynamic behavior of *B. coagulans* during the hierarchical utilization of mixed carbon sources, we performed batch fermentation of *B. coagulans* on the mixture of glucose and trehalose, a disaccharide with a high percentage in the hydrolysates (33). We observed the hierarchical utilization of glucose and trehalose, which divided the entire fermentation process into three phases (Fig. 1A). In the glucose utilization phase (*P*_gluc_, 0–12 h), glucose was exclusively consumed, and lactate was rapidly produced, with the lactate yield on glucose being 0.897 g lactate/g of glucose, which was similar to the yield (i.e., 0.882 g lactate/g of glucose) on glucose as the sole carbon source (Fig. 1B) and close to the yield of homolactic fermentation (28, 34). Upon glucose depletion, the lag phase occurred (*P*_lag_, 12–16 h), in which the lactate production stopped, the biomass concentration (measured by OD_620_) declined (Fig. 1A), and a few organic acids were slightly consumed (Fig. S1). The biomass decline was also observed in the growth profiles on a mixture of glucose and xylose and switchgrass hydrolysate (35). We confirmed that the growth arrest was not caused by the depletion of nutrients such as amino acids (Fig. S2). In the trehalose utilization phase (*P*_tre_, 16–84 h), trehalose was consumed, and lactate was produced again (Fig. 1A). Interestingly, the lactate yield on trehalose in *P*_tre_ was only 0.526 g lactate/g of trehalose, which was much lower than that (i.e., 0.851 g lactate/g of trehalose) on trehalose as the sole substrate (Fig. 1B) and close to the theoretical lactate yield of heterolactic fermentation (28). This was in line with the increased yields of byproducts in *P*_tre_ (Fig. 1B; Fig. S1). The significant change in the lactate yield during the hierarchical utilization of glucose and trehalose indicated distinct strategies to catabolize the carbon sources in *P*_gluc_ and *P*_tre_.

### Dynamic minimization of reallocation of proteome predicted metabolic changes

To gain a systematic understanding, we reconstructed a genome-scale metabolic model of the strain *B. coagulans* DSM 1 = ATCC 7050 (36) called iBcoa620, which was evaluated by the Memote score (37), with a total score of 81% and stoichiometric consistency of 97.7%, and validated by growth on various carbon sources (Fig. S3). Subsequently, we converted iBcoa620 into an enzyme-constrained version, eciBcoa620, using the GECKO toolbox (38), allowing for understanding the metabolic changes by taking into account the enzyme usage and proteome allocation (39–41).

**Fig 2:**
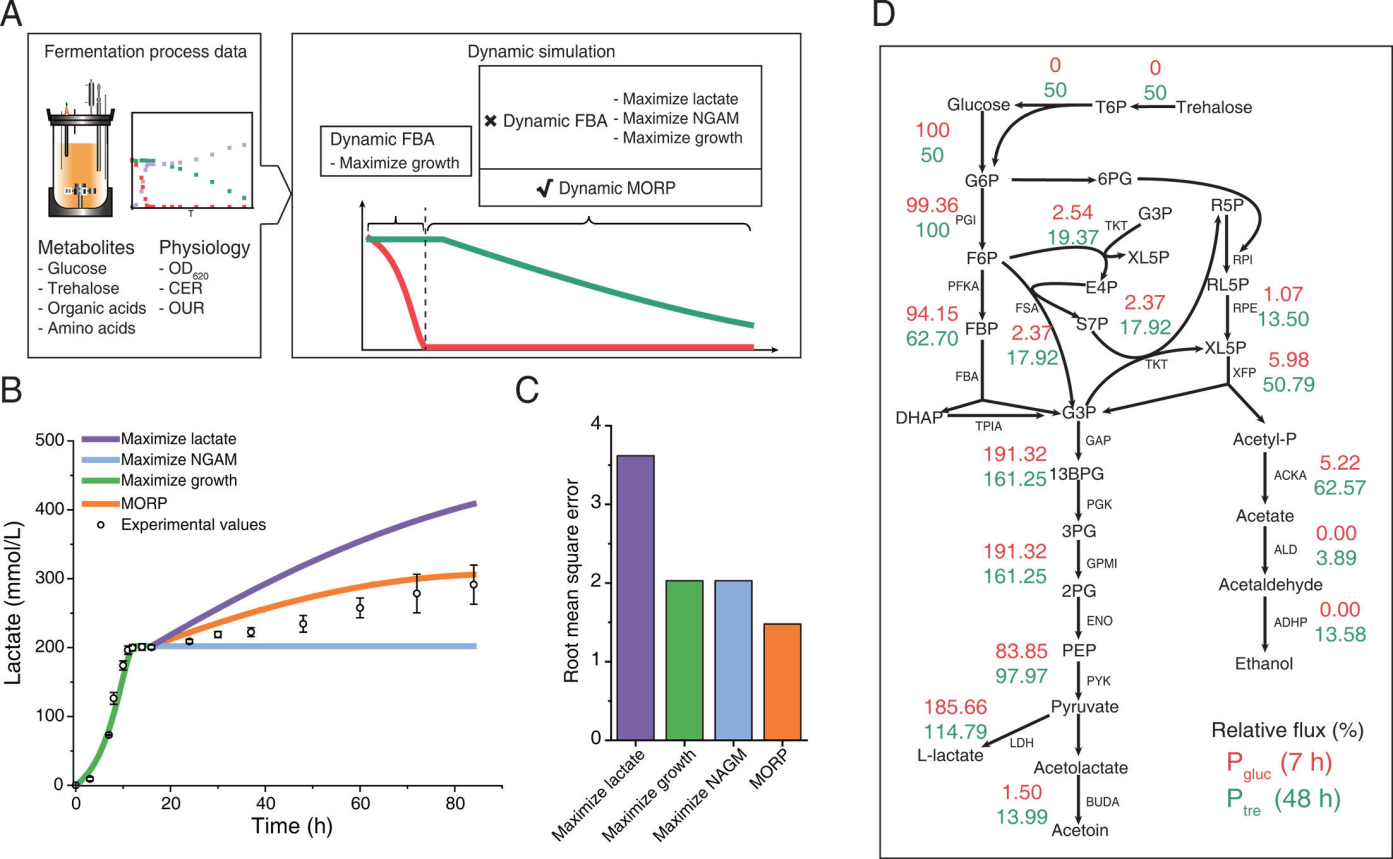
Simulation of batch fermentation under mixed carbon sources by eciBcoa620. (**A**) Simulation of the fermentation process. In the dFBA simulation of *P*_gluc_, the growth rate was maximized. To simulate *P*_tre_, various objective functions could be adopted within the dFBA framework. Additionally, we developed an approach to dynamically minimize the proteome reallocation with the enzyme-constrained models. The red and green lines in the schematic diagram represent the concentration changes of glucose and trehalose, respectively. (**B**) Simulation of lactate production using different objective functions. The lactate production profile for maximizing growth overlaps with that for maximizing NGAM in the trehalose phase. Minimization of proteome reallocation predicted the lactate production in *P*_tre_ better than the others, including maximizing lactate, NGAM, and growth. Data in the plot are shown with mean ± s.d. of three biological replicates. (**C**) Root mean square errors of the different objective functions in predicting *P*_tre_. Root mean square error quantifies the difference between the predicted and experimental concentrations of detected products, including lactate, acetate, pyruvate, acetoin, and ethanol. (**D**) Comparison of the relative fluxes of the central carbon metabolism between *P*_gluc_ (represented by 7 h) and *P*_tre_ (represented by 48 h). Relative flux is calculated as the percentage of the absolute flux of each reaction to the substrate uptake flux. Note that one unit of trehalose uptake flux was converted to two units of glucose uptake flux. T6P, trehalose 6-phosphate; G6P, glucose 6-phosphate; F6P, fructose 6-phosphate; FBP, fructose 1,6-bisphosphate; DHAP, dihydroxyacetone phosphate; G3P, glyceraldehyde 3-phosphate; 13BPG, glycerate 1,3-bisphosphate; 3PG, 3-phosphoglycerate; 2PG, 2-phospho-D-glycerate; PEP, phosphoenolpyruvate; 6PG, gluconate 6-phosphate; E4P, erythrose 4-phosphate; S7P, sedoheptulose 7-phosphate; R5P, ribose 5-phosphate; RL5P, ribulose 5-phosphate; XL5P, xylulose 5-phosphate; Acetyl-P, acetyl phosphate; PGI, glucose-6-phosphate isomerase; PKFA, 6-phosphofructokinase; FBA, fructose-1,6-bisphosphate aldolase; GAP, glyceraldehyde-3-phosphate dehydrogenase; PGK, phosphoglycerate kinase; GPMI, phosphoglycerate mutase; ENO, enolase; PYK, pyruvate kinase; LDH, L-lactate dehydrogenase; RPI, ribose 5-phosphate isomerase; RPE, ribulose-phosphate 3-epimerase; XFP, phosphoketolase; FSA, fructose-6-phosphate aldolase; TKT, transketolase; BUDA, acetolactate decarboxylase; ACKA, acetate kinase; ALD, aldehyde dehydrogenase; and ADHP, alcohol dehydrogenase.

To predict the changes during the fermentation process, we performed dynamic flux balance analysis (dFBA) (42) with eciBcoa620 (Fig. 2A). The dFBA approach can divide the entire period into multiple time intervals and obtain the flux distribution at each time interval by performing classical FBA (31), which requires a predefined objective function. While growth maximization (30) as the objective function appeared reasonable to simulate for *P*_gluc_, it could not predict the cellular behavior after glucose depletion (Fig. 2B), meaning that cells might adjust the objective in response to the changing environment. Therefore, we attempted other commonly used objective functions (43), including maximizing lactate production and non-growth-associated maintenance (NGAM), and found that they also failed to capture the cellular behavior in *P*_tre_ (Fig. S4A), i.e., predicting either much higher lactate concentrations than measurements or no lactate production (Fig. 2B).

Considering the fact that proteome reallocation requires frequent protein synthesis and degradation, which is a high cost to cells (43), we hypothesized that cells tend to dynamically minimize proteome reallocation in response to rapid environmental changes. To test this hypothesis, we developed the MORP approach, which aims to minimize the sum of absolute differences in enzyme usage fluxes between the reference and perturbed conditions. So, the predictive accuracy of MORP relies on precise input parameters derived from preceding time point data. Subsequently, we simulated the glucose phase using dFBA with growth maximization and extended MORP to the dynamic version dMORP to simulate the cellular behavior after glucose depletion, and the enzyme usage in the last time point of the glucose phase was the reference for the first time point for the simulations by dMORP (Fig. 2A) (Materials and Methods). The dMORP approach leverages the enzyme-constrained models and minimizes the sum of absolute differences of all enzyme usage values between previous and current time intervals (Materials and Methods). By employing dMORP, we found that the fermentation phenotype in *P*_tre_ was well predicted (Fig. 2B; Fig. S4B). Moreover, dMORP outperformed other tested objective functions as it led to the smallest root mean square error (Fig. 2C), and it predicted production of byproducts in *P*_tre_ such as acetate, acetoin, and ethanol (Fig. S4A). Furthermore, we compared the metabolic fluxes predicted by various objective functions with those by random sampling, which enables unbiased estimations of metabolic fluxes subject to experimentally measured constraints. For reactions in the central carbon metabolism (CCM), the fluxes predicted by dMORP were generally within the ranges of the sampled fluxes, while the fluxes by other objective functions notably deviated (Fig. S5). Therefore, we demonstrated that dMORP could be a more realistic objective function for describing the cellular behavior after glucose depletion.

**Fig 3:**
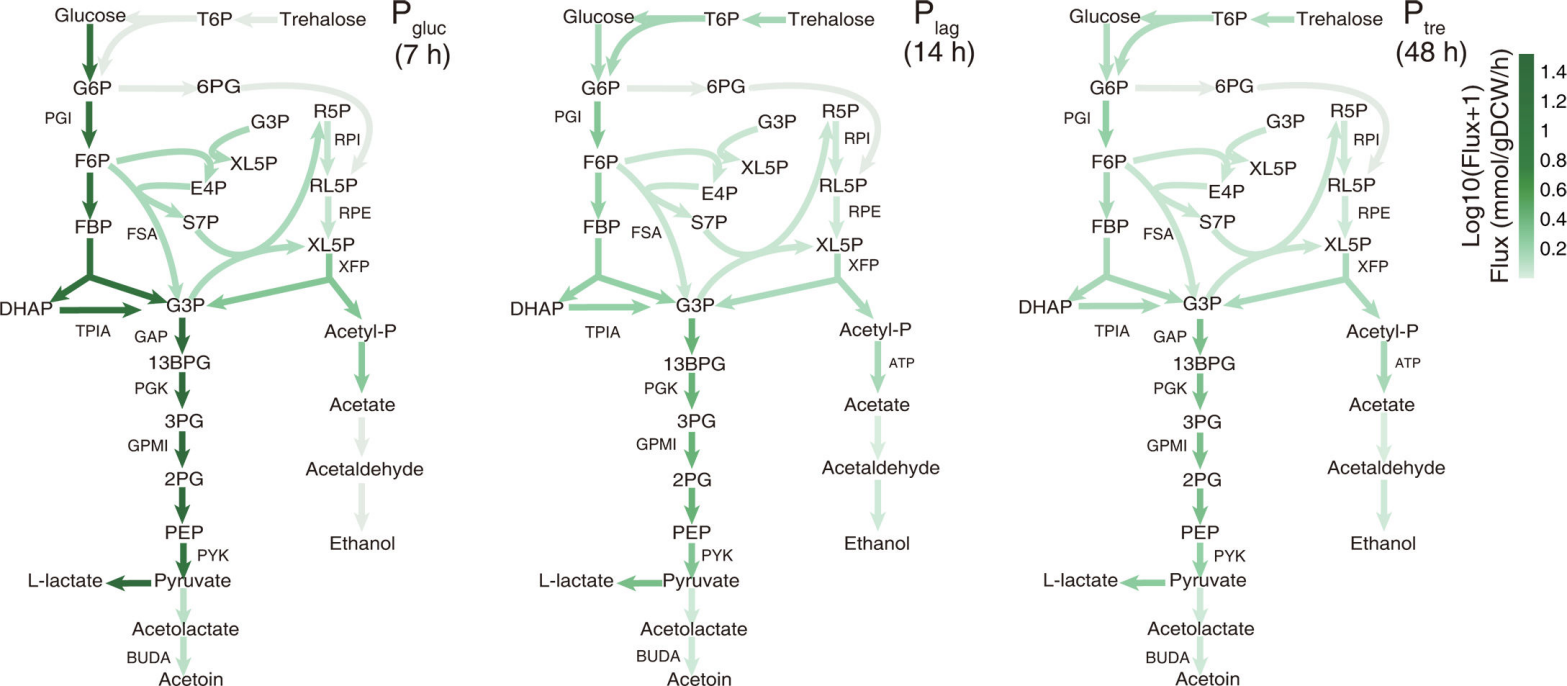
Fluxes of the CCM reactions in the fermentation process predicted by eciBcoa620. The metabolic flux distributions of the CCM at 7 14, and 48 h. These three time points represent *P*_gluc_, *P*_lag_, and *P*_tre_, respectively. At 7 h, the objective function was to maximize growth, while at 14 and 48 h, it was to minimize the proteome reallocation.

To explain the observed lower lactate yield in *P*_tre_ with dMORP, we compared the relative fluxes, i.e., normalized to carbon source uptake, of the CCM reactions in *P*_tre_ (simulated at 48 h, i.e., middle in *P*_tre_) to those in *P*_gluc_ (simulated at 7 h, i.e., mid-exponential phase in *P*_gluc_). In P_tre_, we found a much greater fraction of carbon flux toward phosphoketolase (Fig. 2D), resulting in carbon loss in the form of acetate and ethanol, thereby decreasing the lactate yield (13). This was also similar to the comparison between *P*_tre_ and the sole trehalose condition (Fig. S6). In addition, we compared the absolute fluxes simulated at 7, 14, and 48 h, representing *P*_gluc_, *P*_lag_, and *P*_tre_, respectively (Fig. 3). We found that the metabolic fluxes in *P*_lag_ and *P*_tre_ were comparable and generally less than those in *P*_gluc_ (Fig. 3), exemplified by the glycolytic fluxes, which was due to the considerably slower substrate uptake (Table S1). By comparing *P*_gluc_ and *P*_tre_, we found that the phosphoketolase pathway displayed smaller decreases in fluxes than glycolytic reactions (Fig. S7) and thus accounted for a greater fraction of carbon flux, which explained the higher yields of acetate and ethanol.

### Omics data coincided with dynamic minimization of proteome reallocation

To systematically unravel the molecular mechanisms, we collected transcriptomics and proteomics data at 7, 14, and 48 h based on the fermentation phenotype (Fig. 1A), representing *P*_gluc_, *P*_lag_, and *P*_tre_, respectively. The transcriptomics and proteomics data, which showed high consistency among biological triplicates while clear variability among different phases (Fig. S8), were used for identifying differentially expressed transcripts and proteins (Materials and Methods). Enrichment analysis of the differential expression showed that from *P*_gluc_ to *P*_lag_ and *P*_tre_ pathways were mostly downregulated, particularly those related to translation, e.g., ribosome and aminoacyl-tRNA biosynthesis (Fig. S9), in line with the observed growth arrest (Fig. 1A). Moreover, the differential expression was enriched in amino acid pathways, which could be associated with the significant changes in the concentrations of intracellular amino acids (Fig. S10).

**Fig 4:**
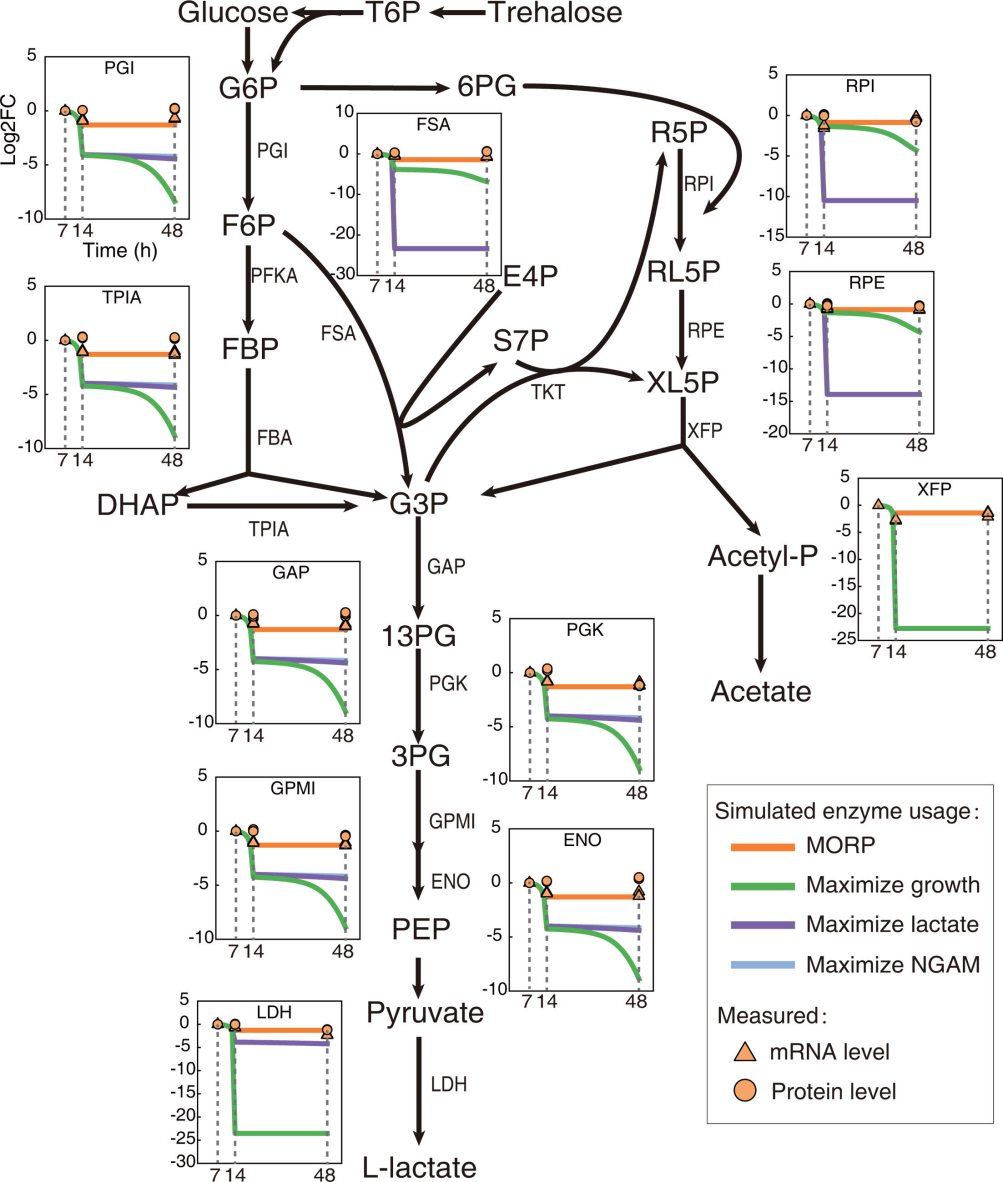
Comparison of simulated enzyme usage and measured mRNA and protein levels. Taking the PGI reaction as an example, the log2FC value represents the change in enzyme usage, mRNA, or protein level relative to the 7 h point. The enzyme usage was predicted by different objective functions. The mRNA and protein levels represent average values of biological triplicates from measured transcriptomics and proteomics data.

Given that the enzyme-constrained models can simulate enzyme usage for metabolic reactions, we compared the simulated enzyme usage to the measured mRNA and protein levels for CCM reactions. We simulated dynamic changes in the enzyme usage using dMORP and dFBA with maximization of growth, lactate, and NGAM and found that dMORP resulted in the least decrease in the enzyme usage upon glucose depletion (Fig. 4), indicating the effectiveness of MORP in minimizing the proteome reallocation during the transition. Notably, the enzyme usage simulated by MORP captured the measured mRNA and protein levels for CCM reactions much better than those simulated by the other objective functions (Fig. 4).

To further compare with MORP, we performed simulations by constraining exchange rates with measurements and minimizing the total proteome (Materials and Methods) and thus estimated the most efficient usage of the enzymes that maintain the experimentally observed metabolic states. This approach, which is widely adopted for enzyme-constrained simulations (38, 44), also performed worse than MORP in terms of simulating the dynamics of the enzyme usage (Fig. S11). In addition, this may indicate suboptimal performance of enzymes during the transition as predicted by MORP (Fig. S12), which could be associated with enzyme occupancy by substrates and thermodynamics (45).

In conclusion, the model simulations and experimental measurements demonstrated that the cellular proteome maintained minimal adjustment upon rapid environmental changes, i.e., after glucose depletion, cells tended to utilize the proteome expressed in the glucose phase to consume trehalose.

### Application of MORP to evolved strains capable of co-utilizing mixed carbon sources

**Fig 5:**
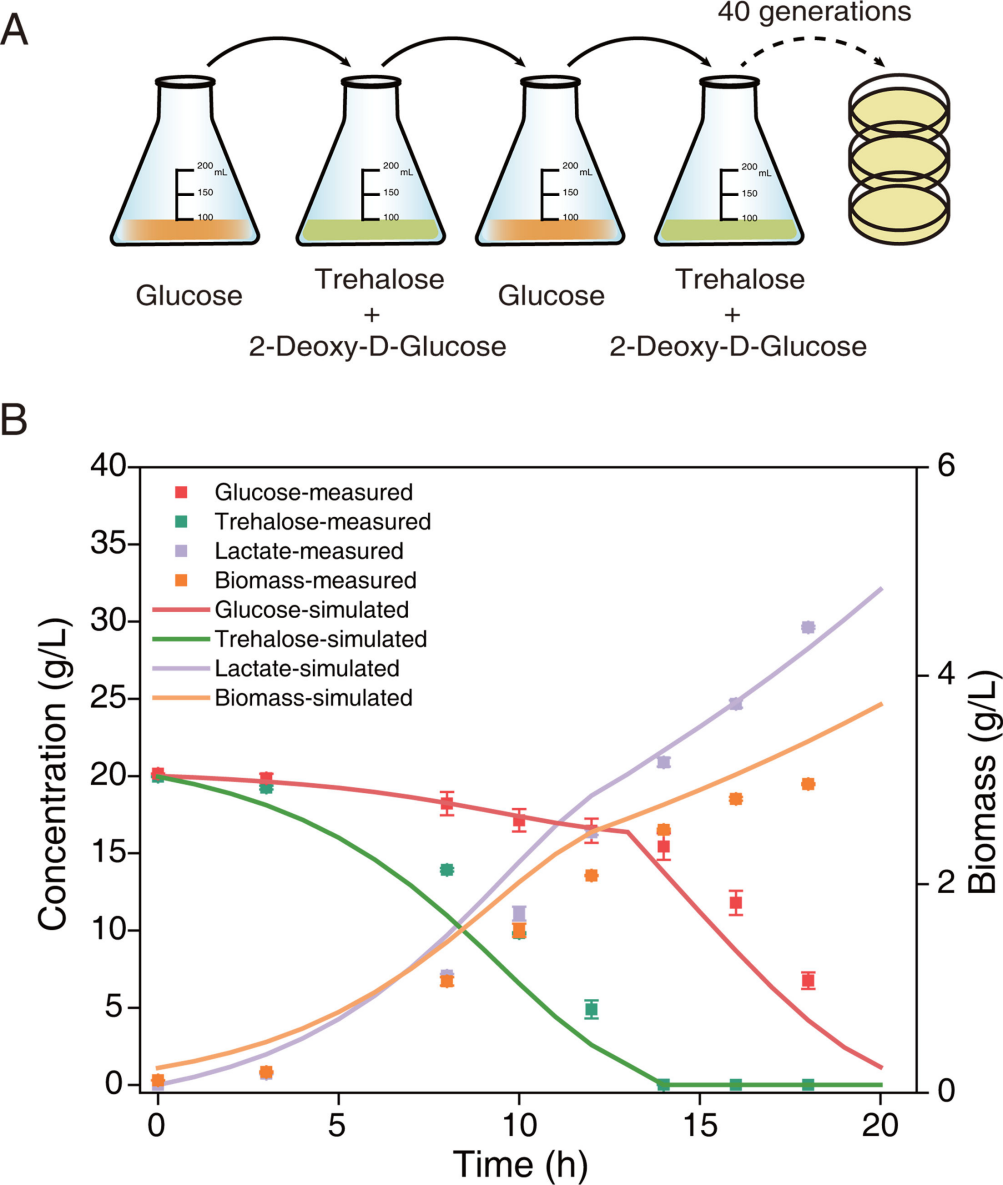
Adaptive laboratory evolution and fermentation process of the evolved strain. (**A**) Brief diagram of the adaptive laboratory evolution experiment. The addition of 2-deoxy-D-glucose to trehalose aimed at efficiently utilizing trehalose in the absence of glucose, with the intention of eliminating the catabolite repression effect. After 40 generations, growth and lactate production were basically stable. The strains with notably improved lactate production were selected for subsequent shake flask validation and 5 L bioreactor experiments. (**B**) Fermentation with mixed carbon sources in a 5 L bioreactor by the evolved strain Ev3. Simulations were performed for the fermentation process, in which dFBA with maximization of growth was performed in the phase of simultaneous utilization of glucose and trehalose, and dMORP was performed after trehalose depletion. Data are shown with mean ± s.d. of three biological replicates.

Given that the hierarchical utilization of carbon sources was accompanied by the metabolic transition from homolactic to heterolactic fermentation, we expected that co-utilization of carbon sources would avoid heterolactic fermentation and thus enhance lactate yield. To this end, we carried out adaptive laboratory evolution to eliminate hierarchical utilization by alternating cultures of glucose and 2-deoxy-glucose + trehalose (Materials and Methods) (Fig. 5A) and obtained four evolved strains that can co-utilize glucose and trehalose (Fig. S13). Whole-genome sequencing identified the mutation in the gene *crr* encoding glucose-specific phosphotransferase enzyme IIA component in all evolved strains (Table S2), in line with the findings of the microbes with diminished glucose repression (46–48).

We grew one of the evolved strains, Ev3, on the mixture of glucose and trehalose in the 5 L bioreactor cultivation and monitored biomass and extracellular metabolite levels (Fig. S14). We found that Ev3 utilized glucose and trehalose simultaneously until 14 h when trehalose was exhausted, and then glucose served as the sole carbon source. By calculating the lactate yield for these two phases, we found that Ev3 performed homolactic fermentation on both phases, which led to a higher total lactate yield than the wild-type strain (Fig. S14).

Furthermore, the dynamics from mixed carbon sources to glucose utilization provided another scenario that could test MORP. To this end, we simulated the fermentation process of Ev3 within the dFBA framework, in which the first phase (before 14 h) was simulated by maximizing growth and the second (after 14 h) by MORP. We showed that MORP could also capture the physiological changes after trehalose depletion (Fig. 5B) and therefore foresee more applications of MORP.

## DISCUSSION

Here, we observed that *B. coagulans* utilized glucose and trehalose hierarchically and transitioned from homolactic to heterolactic fermentation during hierarchical utilization (Fig. 1). We hypothesized that the dynamic minimization of proteome reallocation might result in this observation. To test this hypothesis, we reconstructed the enzyme-constrained genome-scale metabolic model of *B. coagulans* and developed MORP and its dynamic version, dMORP. The model with dMORP captured the transition from homolactic to heterolactic fermentation (Fig. 2) and simulated minimal adjustment of proteome during the transition as indicated by the omics data (Fig. 4). While maximization of biomass is generally an appropriate objective function for simulating both batch and fed-batch fermentations (30), it is unsuitable for modeling carbon source transition systems in this study, as clearly demonstrated by our simulation results (Fig. 2). Although our MORP framework effectively predicted fermentation phenotypes and showed broad qualitative agreement with multi-omics data, some discrepancies between simulated results and measured protein and mRNA levels remain (Fig. S15). These inconsistencies may arise from the current limitations of the model in incorporating protein synthesis and degradation mechanisms (49) and enzyme activity changes (50). In addition, the inconsistencies between protein and mRNA levels may be caused by, e.g., translational regulation (51), post-translational modifications (52), and mRNA degradation processes (53). In summary, we concluded that the dynamic minimization of proteome reallocation could explain the microbial choice of the metabolic strategies upon environmental changes, e.g., changes in the availability of carbon sources in the medium. Finally, with the evolved strain, we used dMORP to simulate the transition from the co-utilization of glucose and trehalose to the utilization of glucose after trehalose depletion (Fig. 5), demonstrating the extended applications of MORP and dMORP.

The biological basis of the minimization of proteome reallocation is the high cost and slow adjustment of protein synthesis and degradation (43, 54, 55) that the cells should consider when adapting to rapid perturbations. Additionally, the minimization of proteome reallocation is in line with the findings of proteome reserves (56, 57), i.e., the proteome is not fully optimized for the environmental condition where the cells are living, as a suboptimal proteome could reduce the cost and time of reallocation upon the adaptation to a new condition. By integrating the minimization of proteome reallocation into the constraint-based modeling framework, we developed MORP and dMORP that introduce the past proteome as an internal constraint, which provides additional objective functions for simulating cellular behaviors (58). Particularly, dMORP would serve as a promising algorithm in the field of dynamic metabolic modeling to predict cellular kinetics (59, 60) and history-dependent behaviors (54, 61, 62).

The approach of minimization of metabolic adjustment (MOMA) has been widely used to simulate cellular behaviors in response to genetic or environmental perturbations (63–65) by minimizing the sum of absolute changes in metabolic fluxes before and after perturbations (66). Compared with minimizing the adjustment of metabolic fluxes, we argue that it is more biologically meaningful to minimize the adjustment of the proteome, which can now be achieved by MORP using enzyme-constrained models. It should be noted that MORP can predict the mismatch between enzyme and flux levels, which appears to be an impossible mission for the MOMA approach implemented on conventional GEMs. In addition, it is less computationally expensive to perform the dynamic simulations on enzyme-constrained models than on fine-grained models, such as ME models (67, 68) that explicitly formulate gene expression processes (23, 69). Therefore, we expect extensive applications of MORP and dMORP with the development of enzyme-constrained models (70).

In summary, we applied systems biology approaches to decipher the principles of the cellular response of *B. coagulans* upon the hierarchical utilization of mixed carbon sources and identified the dynamic minimization of proteome reallocation to be the cellular objective that dominates the metabolic behavior in response to rapid environmental changes. In addition, we implemented the minimization of proteome reallocation as an effective objective function in the metabolic modeling framework, which would be useful for simulating and understanding cellular responses upon perturbations. Furthermore, we believe that MORP can be applied to the simulation of genetic perturbations and guide metabolic engineering. It is also expected that the strains obtained through ALE in the study can improve the carbon source utilization efficiency and lactate production in industrial fermentation.

## MATERIALS AND METHODS

### Microorganism, media, and culture conditions

*B. coagulans* DSM 1 = ATCC 7050 was purchased from the American Type Culture Collection.

Seed medium was MRS medium (71), simplified chemically defined medium used for fermentation in this study, i.e., MCDM3++, which was developed in our previous study (36). Glucose (20 g/L) and trehalose (20 g/L) were used as the mixed carbon source to better reproduce the phenomenon of hierarchical utilization (Table S3).

Seed culture and shake flask fermentation were performed in 250 mL shake flasks with 100 mL working volume at 50°C and 100 rpm. pH was buffered with CaCO_3_. The cells in seed culture were collected by centrifugation at 4°C and 4,000 rpm for around 12 h and washed twice using 25 mL of 100 mM phosphate buffer. Collected cells were resuspended in a 10% fermentation volume of ultrapure water and then transferred to the fermentation medium in shake flasks or 5 L bioreactors. Shake flask fermentation was used to investigate the metabolic properties of different combinations of mixed carbon sources.

Batch fermentation was performed in a 5 L bioreactor with 4 L working volume at 50°C, 100 rpm, and 7.2 L/h aeration, and pH was automatically controlled at 5.5 using 25% (wt/vol) Ca(OH)_2_. The samples of different phases were collected for determining biomass, metabolites, transcriptome, and proteome. Fermentations were performed in triplicate for all conditions.

### Determination of biomass and metabolites

The optical density (OD) was detected using a spectrophotometer at 620 nm to characterize the biomass concentration.

Lactate, acetate, citrate, pyruvate, and acetoin in the fermentation broth were determined by high-performance liquid chromatography (HPLC) (Shimadzu LC‐20AT) (27). The Hi-Plex H (300 × 7.7 mm) column was used with a wavelength of 210 nm and a column temperature of 50°C; the mobile phase was a 0.01 mol/L sulfuric acid solution, and the flow rate was set to 0.4 mL/min. The HPLC detection method for ethanol is as follows: the Aminex HPX-87H (300 × 7.8 mm) column was used with a refractive index detector and a column temperature of 60°C; the mobile phase was a 0.01 mol/L sulfuric acid solution, and the flow rate was set to 0.6 mL/min.

The carbon sources in the fermentation broth were determined by ion chromatography (Dionex ICS-3000) with a Dionex Carbopac PA 20 column. The method was referred to in the previous description (33) and modified. The pretreatment process of the samples was performed as follows: (i) the samples were diluted to achieve sugar concentrations within the detection range. (ii) Na_2_C_2_O_4_ was used to precipitate calcium ions in the culture medium. (iii) Proteins were removed from the samples using ethanol. (iv) The processed solution was evaporated to obtain the solid form of the sugar, which was then dissolved in water to obtain the sample for testing. The mobile phase was ultrapure water and 200 mmol/L NaOH, respectively. The elution was performed using ultrapure water and 200 mmol/L NaOH at a 9:1 ratio for 18 min. The ratio was then adjusted to 2:8 to elute for 17 min and finally adjusted back to 9:1 to balance the column for 35 min. The flow rate for the entire process was set to 0.4 mL/min.

### Reconstruction of genome-scale metabolic models

The model iBcoa620 was reconstructed based on the first *B. coagulans* model iBag597 (13): (i) a combined draft model from KEGG (72) and MetaCyc (73) pathway databases based on the genome sequences of *B. coagulans* DSM 1 = ATCC 7050 (GCF_000832905.1) was reconstructed by RAVEN 2.4.0 (74). (ii) Another draft model was reconstructed by the ModelSEED (75) database based on the genome annotation of RAST (76). (iii) Shared reactions in the draft models were integrated with iBag597. (iv) The IDs of genes, metabolites, and reactions were unified into the form of the KEGG database. (v) Reactions with mass imbalance were corrected. (vi) The memote score system was used to assess the model quality. (vii) The model was validated by comparing the predicted and measured growth rates under different carbon sources (Fig. S3).

The enzyme-constrained model eciBcoa620 was reconstructed using the GECKO toolbox 3.0. Note that *k*_cat_ values were obtained from BRENDA (77) and GotEnzymes (78) databases and predicted using the DLKcat tool (79). Molecular weights of all enzymes were obtained from the UniProt (80) database. All the retrieved data were used for the reconstruction of eciBcoa620.

### Simulation methods

MORP is implemented on the enzyme-constraint models, which can estimate both metabolic and enzyme usage fluxes. MORP estimates the metabolic and enzyme usage fluxes of the model upon a perturbation based on the minimization of proteome reallocation. Thus, the fluxes of the model from a reference condition should be used as the input of MORP, which can be obtained in advance by other algorithms, e.g., FBA, with the enzyme-constraint models. The core of MORP is to minimize the sum of absolute differences in the enzyme usage fluxes between the reference and perturbed conditions:


$$\min\;\;\;\;\;\;\;\;\sum_{i=1}^{m}\left|v_{enzyme,i,perturb}-v_{enzyme,i,ref}\right|$$

 subject to S⋅v=0

$$\;\;\;\;\;\;\;\;\;\;\;\;\;\;\;\;\;\;\;\;\;\;\;\;\;\;\;\;\;\;\;\;\;\;\;\;\;\;\;lb_{j}\leq v_{j}\leq ub_{j},$$

where *v*_enzyme,*i*,perturb_ and *v*_enzyme,*i*,ref_ represent fluxes of enzyme usage reaction *i* under perturbed and reference conditions, and *m* represents the total number of enzyme usage reactions in the model. As MORP estimates fluxes for the perturbed condition, ***S*** and ***v*** represent the stoichiometric matrix and flux vector of the model for the perturbed condition, and *v*_*j*_, *lb*_*j*_, and *ub*_*j*_ are the flux, lower, and upper bounds of reaction *j*, respectively.

dFBA based on a static optimization approach (81) was used to simulate the hierarchical utilization of glucose and trehalose, in which an objective function should be determined for different time intervals. As mentioned before, three objective functions were used, i.e., maximization of growth, lactate production, and NGAM, with eciBcoa620. Generally, dFBA solves


$$\max\;\;\;\;\;\;\;\;\;v_{o,k}\;\;\;\;k\in N$$

 subject to S⋅v=0

$$\;\;\;\;\;\;\;\;\;\;\;\;\;\;\;\;\;\;\;\;\;\;\;\;\;\;\;\;\;\;\;\;\;\;\;\;\;\;\;\;\;\;v_{s,k}=\frac{v_{s,max}{\left[S\right]}_{k-1}}{K_{m}+{\left[S\right]}_{k-1}},$$

$$\;\;\;\;\;\;\;\;\;\;\;\;\;\;\;\;\;\;\;\;\;\;\;\;\;\;\;\;\;\;\;\;\;lb_{j}\leq v_{j}\leq ub_{j},$$

where *k* represents the *k*_th_ time interval, *N* represents the number of time intervals of the phase of interest and thus is a positive integer, *v*_*o,k*_ is the flux of the objective reaction in *P*_gluc_, i.e., the biomass formation, or the flux of the objective reaction after glucose depletion, i.e., the biomass formation, lactate production, or NGAM production, *v*_*s,k*_ is the flux of the uptake reaction of the sugar, i.e., glucose or trehalose, of the kth time interval, [*S*]_*k*-1_ is the sugar concentration of the (*k* − 1)_th_ time interval, *v*_*s*,max_ is the maximum sugar uptake rate, and *K*_*m*_ is the sugar saturation constant. The constraints in dFBA were derived from experimentally measured sugars, amino acids, and organic acid data.

dMORP was proposed to simulate the entire period after glucose depletion in the hierarchical utilization of glucose and trehalose. Here, dMORP solves:


$$\min\;\;\;\;\;\;\;\;\sum_{i=1}^{m}\left|v_{enzyme,i,r}-v_{enzyme,i,r-1}\right|\;\;\;\;\;r\in N$$

 subject to S⋅v=0

$$\;\;\;\;\;\;\;\;\;\;\;\;\;\;\;\;\;\;\;\;\;\;\;\;\;\;\;\;\;\;\;\;\;\;\;\;\;\;\;\;\;\;v_{s,r}=\frac{v_{s,max}{\left[S\right]}_{r-1}}{K_{m}+{\left[S\right]}_{r-1}},$$

$$\;\;\;\;\;\;\;\;\;\;\;\;\;\;\;\;\;\;\;\;\;\;\;\;\;\;\;\;\;\;\;\;\;\;lb_{j}\leq v_{j}\leq ub_{j},$$

where *r* represents the *r*_th_ time interval after glucose depletion, *N* represents the number of time intervals of the phase of interest and thus is a positive integer, *v*_enzyme,*i*,*r*_ and *v*_enzyme,*i*,*r*-1_ represent the flux of enzyme usage reaction *i* for the *r*_th_ and (*r* − 1)_th_ time interval, respectively, *v*_*s*,*r*_ is the flux of the trehalose uptake reaction of the *r*_th_ time interval, and [*S*]_*r*-1_ is the trehalose concentration of the (*r* − 1)_th_ time interval.

Minimization of the total proteome was performed to estimate the most efficient usage of the enzymes that maintain the experimentally observed metabolic states:


$$min\;v_{total\;proteome}$$

 subject to S⋅v=0

$$\;\;\;\;\;\;\;\;\;\;\;\;\;\;\;\;\;\;\;\;\;\;\;\;\;\;\;\;\;\;\;\;\;\;lb_{i}\leq v_{i}\leq ub_{i},$$

where *v*_total proteome_ represents the flux of total proteome usage, *v*_*i*_ represents the flux of exchange reaction of detected extracellular metabolite, and *lb*_*i*_ and *ub*_*i*_ are lower and upper bounds based on measurements.

All the simulations were performed with the COBRA toolbox 3.0 (82) on Matlab R2019a, and the solver was Gurobi 9.1 (https://www.gurobi.com/).

### Transcriptome analysis

We collected transcriptomics samples at 7, 14, and 48 h, representing *P*_gluc_, *P*_lag_, and *P*_tre_, respectively. Cells in the fermentation broth were collected by centrifugation at 4°C and 4,000 rpm and washed three times with phosphate-buffered saline for subsequent RNA extraction. Total RNA was isolated from cells using TRIzol Reagent following the instructions, and genomic DNA was removed using DNase I (TaKara). Subsequently, RNA quality was determined using an Agilent 2100 Bioanalyzer and quantified using ND-2000 (NanoDrop Technologies).

RNA library construction was performed using the TruSeqTM RNA sample preparation Kit from Illumina (San Diego, CA, USA). The rRNA was removed using the Ribo-Zero Magnetic kit (Epicenter), and double-stranded cDNA was reverse transcribed using random primers (Illumina) and SuperScript Double-Stranded cDNA Synthesis Kit (Invitrogen, CA, USA). To synthesize the second strand of cDNA, it uses dUTP instead of dTTP and then eliminates the second strand of cDNA containing dUTP, so that the library contains only the first strand of cDNA. After PCR amplification by Phusion DNA polymerase (NEB) and TBS380 (Picogreen) quantification, the paired-end RNA-seq sequencing library was sequenced with the Illumina Novaseq (2 × 150 bp read length).

The data generated from the Illumina platform were used for bioinformatics analysis, and the reference genome and gene annotation files of *B. coagulans* DSM 1 = ATCC 7050 were downloaded from the National Center for Biotechnology Information. All the analyses were performed using the free online platform of Majorbio Cloud Platform (https://www.majorbio.com/).

### Proteome analysis

We collected proteomics samples at 7, 14, and 48 h, representing *P*_gluc_, *P*_lag_, and *P*_tre_, respectively. Cells in the fermentation broth were collected by centrifugation at 4°C and 4,000 rpm and washed three times with phosphate-buffered saline for subsequent protein extraction. Sample lysis and protein extraction were performed using SDT (4% SDS, 100 mM Tris-HCl, and 1 mM DTT, pH 7.6) buffer, and the protein was quantified using the BCA Protein Assay Kit (Bio-Rad, USA). Protein digestion was performed with trypsin following the filter-aided sample preparation procedure. The digested peptides from each sample were subsequently desalted (Empore SPE Cartridges C18 [standard density], bed I.D. 7 mm, volume 3 mL, Sigma) and concentrated. The protein for each sample was quantified by SDS-PAGE. One hundred micrograms of peptide mixture for each sample was labeled using TMT reagent according to the instructions (Thermo Scientific). The labeled peptides were separated by High pH Reversed-Phase Peptide Fractionation Kit (Thermo Scientific) to obtain 10 different fractions. The collected fractions were desalted on C18 Cartridges and concentrated.

LC-MS/MS analysis was performed using a Thermo Scientific Q Exactive mass spectrometer equipped with the Easy nLC system (Proxeon Biosystems, Thermo Fisher Scientific). Peptides were trapped on a reverse phase trap column (Thermo Scientific Acclaim PepMap100, 100 µm × 2 cm, nanoViper C18) and separated on the C18-reversed phase analytical column (Thermo Scientific Easy Column, 10 cm long, 75 µm inner diameter, and 3 µm resin). The separation was achieved using buffer A (0.1% formic acid) and buffer B (84% acetonitrile and 0.1% formic acid) at a constant flow rate of 300 nL/min controlled by IntelliFlow technology. The mass spectrometer was operated in positive ion mode. MS/MS analysis was performed using the data-dependent top 10 method for high-energy collision dissociation fragmentation by selecting the most abundant precursor ion (300–1,800 *m/z*) in the measurement scan. The MS raw data were subjected to identification and quantitative analysis using the MASCOT engine (Matrix Science, London, UK; version 2.2) and Proteome Discoverer 1.4 software. The proteome detection and analysis were conducted in Shanghai Applied Protein Technology Co., Ltd.

### Differential expression analysis

Significance analysis between two conditions or phases was performed using the two-sided *t*-test, and a cutoff adjusted *P* value (*P*_adjusted_) of 0.01 was adopted for identifying differentially expressed transcripts and proteins based on transcriptomics and proteomics data.

### Determination of intracellular metabolites

The absolute quantification of intracellular amino acids was performed using gas chromatography-mass spectrometry (Agilent, GC-7890A, MS-5975C). Before the extraction of intracellular metabolites, a quenching step was performed to terminate cell metabolism. Two milliliters of fermentation broth was added to a 50 mL centrifuge tube containing 30 mL of 60% methanol pre-cooled to −27°C. After quenching for 5 min, the filter paper with attached cells was obtained by filtration. Simultaneously, 100 µL of U-^13^C-labeled cell extracts was added to the filter paper as internal standards. The filter paper was then placed in a 75% ethanol solution pre-heated to 80°C and boiled for 5 min. The filter paper was removed, and the remaining liquid was concentrated to 600 µL using rapid evaporation. A 100 µL portion was taken as the sample and subjected to freeze-drying before derivatization (13, 83, 84).

### Amino acid derivatization method

One hundred microliters of pyridine was added to the freeze-dried sample, and the mixture was dissolved at 65°C for 1 h. After the sample reached room temperature, 100 µL of derivatization reagent (N‐tert‐butyldimethylsilyl‐N-methyl-trifluoroacetamide with 1% tert‐butyldimethylchlorosilane) was added, and derivatization was carried out in a 65°C oven for 1 h. The sample was then centrifuged at 12,000 rpm for 5 min, and the supernatant was subjected to GC-MS analysis.

The GC-MS detection conditions were as follows: the interface temperature of the GC-MS was set at 280°C, and the transfer line temperature was 250°C. Ionization was done by electron impact, with the temperature being 230°C, the quadrupole temperature was 150°C, and the injection volume was 1 µL. The chromatographic column used was HP-5MS with dimensions of 30 mm × 0.25 mm × 0.25 µm. For amino acids, the temperature program consisted of an initial column temperature of 100°C for 1 min, followed by an increase at a rate of 10°C/min to reach 320°C, which was then maintained for 10 min.

### Adaptive laboratory evolution

The adaptive laboratory evolution strategy was performed in a 250 mL shake flask with a complex medium at 100 rpm and 50°C. Alternating cultures of glucose and trehalose were utilized, with the trehalose supplemented with a glucose structural analog (2-deoxy-D-glucose) that can be absorbed but not metabolized. Therefore, other sugars are still able to be metabolized even in the presence of glucose, so 2-deoxy-D-glucose can be used for screening mutant strains without catabolite repression effect (85, 86). The aim was to obtain strains that could utilize glucose and trehalose simultaneously. During the evolution process, the cells were transferred to fresh medium in the exponential phase and maintained the same concentration (OD_620_ about 0.5) after inoculation. The initial total sugar concentration during evolution was kept constant at 130 g/L (glucose 130 g/L, 2-deoxy-D-glucose 40 g/L + trehalose 90 g/L). A concentration of 40 g/L of 2-deoxy-D-glucose was selected as no growth occurred at higher concentrations. Four evolved strains with the expected phenotypes were examined by MRS plate culture, and the strain with the fastest sugar consumption rate and highest lactate yield was selected for fermentation experiments in a 5 L bioreactor.

### Whole-genome sequencing

Cells from the exponential phase in seed shake flasks were collected for genome sequencing. Genomic DNA of *B. coagulans* DSM 1 = ATCC 7050 was extracted using the Wizard Genomic DNA Purification Kit (Promega) according to the protocol. Purified genomic DNA was quantified by TBS-380 fluorometer (Turner BioSystems Inc., Sunnyvale, CA, USA). Whole-genome sequencing of the wild-type strain and four evolved strains was performed using the *de novo* sequencing method based on the Illumina NovaSeq6000 platform in this study. The final genome of five strains was annotated from KEGG, GO, COG, and other databases using BLASTP, Diamond, and HMMER. The same mutated genes of the four evolved strains were considered to be genes that could cause phenotypic change.

## Data Availability

The genome sequences of four evolved strains have been deposited in the NCBI database under the BioProject accession number PRJNA1012373. The transcriptomics data have been deposited in the NCBI database under the BioProject accession number PRJNA1012495. The mass spectrometry proteomics data sets have been deposited in the ProteomeXchange with the data set identifier PXD045169. The models and code are available at https://github.com/ChenYuGroup/MORP.

## SUPPLEMENTAL MATERIAL

Supplemental material msystems.00690-25-s0001.pdf Supplemental figures and tables.

ASM does not own the copyrights to Supplemental Material that may be linked to, or accessed through, an article. The authors have granted ASM a non-exclusive, world-wide license to publish the Supplemental Material files. Please contact the corresponding author directly for reuse.
